# Supplementary material for: Analysis of PPARγ Signaling Activity in Psoriasis
Source: Int J Mol Sci. 2021 Aug 10;22(16):8603. doi: 10.3390/ijms22168603 (PMC8395241; doi:10.3390/ijms22168603)
Supplement: Supplementary file 1 [file ijms-22-08603-s001.zip › Supplemental materials_Analysis of PPARg signaling activity in psoriasis/Pathway models/Models images and html files/Model of PPARG related pathways in psoriasis (short version)/9895.html]

RHEB


# Protein RHEB

|  |  |
| --- | --- |
| URN | urn:agi-llid:6009 |
| Total Entities | 0 |
| Connectivity | 911 |
| Name | RHEB |
| Description | Ras homolog enriched in brain |
| Notes | This gene is a member of the small GTPase superfamily and encodes a lipid-anchored, cell membrane protein with five repeats of the RAS-related GTP-binding region. This protein is vital in regulation of growth and cell cycle progression due to its role in the insulin/TOR/S6K signaling pathway. The protein has GTPase activity and shuttles between a GDP-bound form and a GTP-bound form, and farnesylation of the protein is required for this activity. Three pseudogenes have been mapped, two on chromosome 10 and one on chromosome 22. [provided by RefSeq, Jul 2008] |
| ObjectType | Protein |

---

|  |  |
| --- | --- |
| Pathway | Oncogene Induced Cellular Senescence |
|  | DNA Persistent Repair Inhibits mTOR Signaling |
|  | mTOR Signaling Activation by Fatty Acids and Glucose |
|  | mTOR Signaling Activation by Amino Acids |
|  | mTOR Signaling |
|  | mTOR Signaling Hyperfunction |
|  | Hypoxia Inhibits mTOR Signaling |
|  | SIRT1 Signaling in Aging |
|  | mTOR Signaling Activation by Taste Sensor Receptors (Rodent Model) |
|  | Insulin Influence on Protein Synthesis |
|  | Insulin Signaling |
|  | Proteins Involved in Myocardial Ischemia |
|  | Preconditioning Ischemia |
|  | Glucocorticoids in Insulin Resistance in Skeletal Muscles |
|  | Peutz-Jeghers Syndrome |
|  | Ketogenic Diet Mechanism (Hypothesis) |
|  | Blood-Brain Barrier Disruption in Epileptiform Disorders |
|  | mTOR Hyperactivation after Status Epilepticus |
|  | Angiogenesis in Endometriosis |
|  | Polycystins Mutation Effects in Cystic Kidney Disease |
|  | AREG -> HIF1A Expression Targets |
|  | EGF -> HIF1A Expression Targets |
|  | EGFR/ERBB2 -> HIF1A Signaling |
|  | EREG -> HIF1A Expression Target |
|  | HBEGF -> HIF1A Expression Targets |
|  | N2 Neutrophils in Tumor-Promoting Inflammation and Tumor Progression |
|  | TGFB Signaling activation by Blocking of Tumor Suppressors |
|  | PI3K/AKT/MTOR Signaling Activation by Blocking of Tumor Suppressors |
|  | HIF1A as Master Regulator of Angiogenesis in Cancer |
|  | MTOR/TORC in Cancer Cell Motility and Invasion |
|  | TGFB Family in Epithelial to Mesenchymal Transition in Cancer |
|  | Metabolic Effects of Oncogenes and Tumor Suppressor in Cancer Cells |
|  | NRG1 -> HIF1A Expression Target |
|  | Androgen Receptor non-Genomic Signaling |
|  | TGF-beta Signaling |
|  | AMPK Signaling |
|  | mTOR Signaling |
|  | VEGF Signaling |
|  | EGFR Signaling |
|  | TGFA -> HIF1A Expression Targets |
|  | ace2 |
|  | Model of PPARG signaling in psoriasis |
|  | Model of PPARG related pathways in psoriasis (short version) |
|  | 1\_Formation of cysts in polycystic kidney disease |
|  | 3\_Enhanced angiogenesis in endometriosis |
|  | 2\_Neuron and Schwann cell death |

---

|  |  |
| --- | --- |
| MedScan ID | 6009 |

---

|  |  |
| --- | --- |
| LocusLink ID | 6009 |
|  | 19744 |
|  | 26954 |

---

|  |  |
| --- | --- |
| Alias | GTP-binding protein Rheb |
|  | GTPase Rheb |
|  | Ras homolog enriched in brain 2 |
|  | RAS-homolog enriched in brain |
|  | Rheb guanosine triphosphatase |
|  | OTTMUSP00000027651 |
|  | Rhebs |
|  | Ras homolog enriched in brain II |
|  | Rheb1 |
|  | RHEB |
|  | RHEB2 |
|  | Rheb GTP binding protein |
|  | Rheb GTPases |
|  | Rheb GTPase |
|  | Ras homologue enriched in brain protein |
|  | MGC111559 |
|  | Ras homolog, mTORC1 binding |
|  | Ras homolog enriched in brain |

---

|  |  |
| --- | --- |
| GO ID | 0019003 |
|  | 0005525 |
|  | 0003924 |
|  | 0000287 |
|  | 0019901 |
|  | 0007050 |
|  | 0120163 |
|  | 0032008 |
|  | 0048714 |
|  | 0016241 |
|  | 2000074 |
|  | 0007165 |
|  | 0007264 |
|  | 0000139 |
|  | 0005829 |
|  | 0005789 |
|  | 0070062 |
|  | 0005765 |
|  | 0016020 |
|  | 0005886 |
|  | 0014069 |
|  | 0005681 |
|  | 0046872 |
|  | 0000166 |
|  | 0032006 |
|  | 0099175 |
|  | 0005794 |
|  | 0005737 |
|  | 0030425 |
|  | 0005783 |
|  | 0098978 |
|  | 0043025 |
|  | 0048168 |
|  | 0016236 |
|  | 0015031 |
|  | 0008286 |
|  | 0005622 |

---

|  |  |
| --- | --- |
| KEGG ID | hsa:6009 |
|  | mmu:19744 |
|  | rno:26954 |

---

|  |  |
| --- | --- |
| Organism | Homo sapiens {Organism urn:agi-taxid:9606} |
|  | Mus musculus {Organism urn:agi-taxid:10090} |
|  | Rattus norvegicus {Organism urn:agi-taxid:10116} |
|  | Homo sapiens |
|  | Mus musculus |
|  | Rattus norvegicus |

---

|  |  |
| --- | --- |
| Mouse chromosome position | 5 |

---

|  |  |
| --- | --- |
| OMIM ID | 601293 |

---

|  |  |
| --- | --- |
| Rat chromosome position | 4q11 |
|  | 4 |

---

|  |  |
| --- | --- |
| Hugo ID | 10011 |
|  | HGNC:10011 |

---

|  |  |
| --- | --- |
| Human chromosome position | 7q36.1 |
|  | 7q36 |

---

|  |  |
| --- | --- |
| Swiss-Prot Accession | A0A090N900 |
|  | Q15382 |
|  | Q15382.1 |
|  | Q540E6 |
|  | Q921J2 |
|  | Q921J2.1 |
|  | Q62639 |
|  | Q62639.1 |
|  | B3KWN6 |
|  | D3DX13 |
|  | Q53Y56 |
|  | Q99444 |

---

|  |  |
| --- | --- |
| PIR ID | S68419 |
|  | I55401 |

---

|  |  |
| --- | --- |
| GenBank ID | NC\_000007 |
|  | NM\_005614 |
|  | NP\_005605 |
|  | XM\_011516457 |
|  | XP\_011514759 |
|  | XM\_024446854 |
|  | XP\_024302622 |
|  | AACC02000108 |
|  | EAL24515 |
|  | AC005996 |
|  | AAD15548 |
|  | CH471173 |
|  | EAW53986 |
|  | EAW53987 |
|  | EAW53988 |
|  | EAW53989 |
|  | JC006890 |
|  | CDL93493 |
|  | KT584052 |
|  | AF148645 |
|  | AAF73125 |
|  | AF493921 |
|  | AAM12635 |
|  | AK125446 |
|  | BAG54198 |
|  | AK289370 |
|  | BAF82059 |
|  | BC016155 |
|  | AAH16155 |
|  | BC066307 |
|  | AAH66307 |
|  | BC107705 |
|  | AAI07706 |
|  | BG702213 |
|  | BM670621 |
|  | BT006958 |
|  | AAP35604 |
|  | D78132 |
|  | BAA11211 |
|  | DA475169 |
|  | Z29677 |
|  | CAA82774 |
|  | Q15382 |
|  | NC\_000071 |
|  | NM\_053075 |
|  | NP\_444305 |
|  | AC125270 |
|  | AC137938 |
|  | CH466586 |
|  | EDL03089 |
|  | EDL03090 |
|  | JC006888 |
|  | CDL93492 |
|  | AB039919 |
|  | BAB13483 |
|  | AK151213 |
|  | BAE30208 |
|  | AK158213 |
|  | AY197373 |
|  | AAO43226 |
|  | BC012273 |
|  | AAH12273 |
|  | Q921J2 |
|  | NC\_005103 |
|  | NM\_013216 |
|  | NP\_037348 |
|  | AC\_000072 |
|  | AABR07059178 |
|  | AABR07059179 |
|  | AABR07059180 |
|  | AABR07059181 |
|  | AAHX01027281 |
|  | AAHX01027282 |
|  | CH474057 |
|  | EDL86374 |
|  | EDL86375 |
|  | EDL86376 |
|  | EDL86377 |
|  | BC088857 |
|  | AAH88857 |
|  | FQ212847 |
|  | FQ214530 |
|  | FQ218725 |
|  | FQ219039 |
|  | FQ225701 |
|  | FQ230203 |
|  | U08227 |
|  | AAA21380 |
|  | Q62639 |
|  | NC\_018918 |
|  | AMYH02017074 |
|  | AC\_000027 |
|  | AAHY01042997 |
|  | AAHY01042998 |
|  | AAHY01042999 |
|  | XM\_006716081 |
|  | XP\_006716144 |
|  | AC\_000139 |
|  | ABBA01035154 |
|  | BC009638 |
|  | XM\_006535647 |
|  | XP\_006535710 |

---

|  |  |
| --- | --- |
| Swiss-Prot ID | RHEB\_HUMAN |
|  | RHEB\_MOUSE |
|  | RHEB\_RAT |

---

|  |  |
| --- | --- |
| Cell Localization | Endomembrane system |
|  | Golgi apparatus membrane |
|  | Cytoplasm |
|  | Cytosol |
|  | Endoplasmic reticulum membrane |
|  | Cytoplasm, cytosol |

---

|  |  |
| --- | --- |
| Ensembl ID | ENSG00000106615 |
|  | ENSP00000262187.5 |
|  | ENST00000262187.10 |
|  | ENSMUSG00000028945 |
|  | ENSMUSP00000030787.8 |
|  | ENSMUST00000030787.8 |
|  | ENSRNOG00000050578 |
|  | ENSRNOP00000063915.2 |
|  | ENSRNOT00000071737.2 |
|  | ENSP00000262187 |
|  | ENST00000262187 |
|  | ENSMUSP00000030787 |
|  | ENSMUST00000030787 |
|  | ENSRNOP00000063915 |
|  | ENSRNOT00000071737 |

---

|  |  |
| --- | --- |
| MGI ID | MGI:97912 |

---

|  |  |
| --- | --- |
| RGD ID | 621840 |

---

|  |  |
| --- | --- |
| Unigene ID | Hs.283521 |
|  | Hs.568850 |
|  | Hs.647068 |
|  | Mm.319175 |
|  | Rn.859 |

---

|  |  |
| --- | --- |
| Homologene ID | 123916 |

---
